# 2120. Activity of a Hypochlorous Acid-Generating Electrochemical Bandage for Treating Experimental Murine Wounds Infected with *Pseudomonas aeruginosa*

**DOI:** 10.1093/ofid/ofad500.1743

**Published:** 2023-11-27

**Authors:** Derek Fleming, Dilara Özdemir, Judith Alvarez Ortero, Md Monzurul Anoy, Melissa J Karau, Ibrahim Bozyel, Audrey N Schuetz, Kerryl E Greenwood-Quaintance, Haluk Beyenal, Robin Patel

**Affiliations:** Mayo Clinic, Rochester, Minnesota; Washington State University, Pullman, Washington; Mayo Clinic, Rochester, Minnesota; Washington State University, Pullman, Washington; Mayo Clinic, Rochester, Minnesota; Washington State University, Pullman, Washington; Mayo Clinic, Rochester, Minnesota; Mayo Clinic, Rochester, Minnesota; Washington State University, Pullman, Washington; Mayo Clinic, Rochester, Minnesota

## Abstract

**Background:**

Biofilms formed by antibiotic-resistant bacteria in wound beds present unique challenges in terms of treating wound infections. In this work, the *in vivo* anti-biofilm activity of a novel electrochemical bandage (e-bandage) designed to continuously deliver low amounts of hypochlorous acid (HOCl) was evaluated against wound biofilm infections caused by a clinical isolate of *Pseudomonas aeruginosa* resistant to all antibiotic classes except aminoglycosides.

**Methods:**

5 mm skin wounds were created on the dorsal surface of Swiss-Webster mice and infected with 10^6^ CFU of *P. aeruginosa*. Biofilms were allowed to form over 2 days, after which e-bandages with Tegaderm™ or Tegaderm only were placed on the wound beds. Specifically, mice were randomized to one of three experimental groups (n = 7-8): 1) Tegaderm only (control); 2) non-polarized e-bandage; 3) polarized e-bandage using a wearable potentiostat. After 48 hours, animals were sacrificed and 10 mm skin biopsies containing the wound and surrounding tissue were harvested for bacterial quantification. Histopathology was performed on H&E-stained sections of wound beds from each treatment group. Blood was screened with an inflammatory cytokine panel. Wound healing (% area reduction; digital measurement) and purulence (observational scoring) were assessed in pre- and post-treatment wounds.

**Results:**

48 hours of polarized e-bandage treatment resulted in a mean reduction of 1.86 log_10_ biofilm CFUs/g (p < 0.0001) *vs* non-polarized controls, and 2.18 log_10_ CFU/g reduction (p < 0.0001) *vs* Tegaderm only controls (polarized, 7.6 ± 0.66 log_10_ CFU/g; non-polarized, 9.45 ± 0.26 log_+10_ CFU/g; Tegaderm only, 9.78 ± 0.16 log_10_ CFU/g). Compared to Tegaderm only, purulence reduction and wound healing were significantly higher for both non-polarized (purulence - p < 0.0001; healing - p < 0.01 ) and polarized treatment (purulence - p < 0.0001; healing - p < 0.0001) groups, but were not significantly different from each other. Histopathology and blood inflammation panel testing showed no significant differences between groups.

**Conclusion:**

An HOCl-producing e-bandage reduced *P. aeruginosa* in wound biofilms, representing a potential antibiotic-free strategy for the treatment of recalcitrant wound infections.

**Disclosures:**

**Audrey N. Schuetz, MD**, Merck: Advisor/Consultant **Robin Patel, MD**, Abbott Laboratories: Advisor/Consultant|Adaptive Phage Therapeutics: Grant/Research Support|Adaptive Phage Therapeutics: Mayo Clinic has a royalty-bearing know-how agreement and equity in Adaptive Phage Therapeutics.|BIOFIRE: Grant/Research Support|CARB-X: Advisor/Consultant|ContraFect: Grant/Research Support|Day Zero Diagnostics: Advisor/Consultant|HealthTrackRx: Advisor/Consultant|Mammoth Biosciences: Advisor/Consultant|Netflix: Advisor/Consultant|Oxford Nanopore Technologies: Advisor/Consultant|PhAST: Advisor/Consultant|See details: Patent on Bordetella pertussis/parapertussis PCR issued, a patent on a device/method for sonication with royalties paid by Samsung to Mayo Clinic|See details: continued, patent on an anti-biofilm substance issued|TenNor Therapeutics Limited: Grant/Research Support|Torus Biosystems: Advisor/Consultant|Trellis Bioscience, Inc.: Advisor/Consultant

